# Trends in the Incidence of Rare Primary Cardiac Neoplasms: A Surveillance, Epidemiology, and End Results (SEER)-Based Analysis

**DOI:** 10.7759/cureus.92613

**Published:** 2025-09-18

**Authors:** Joud K Alhousani, Ahmed Abdelmageed, Hamza Khoursheed, Said Yaseen, Mohammad Hamad, Hussam Askari, Leen Abu Rabi, Moh'd Taha, Abdulrahman Barakat, Abdulqadir J Nashwan

**Affiliations:** 1 School of Medicine, Jordan University of Science and Technology, Irbid, JOR; 2 Faculty of Medicine, Mansoura University, Mansoura, EGY; 3 School of Medicine, University of Jordan, Amman, JOR; 4 Nursing and Midwifery Research Department, Hamad Medical Corporation, Doha, QAT

**Keywords:** epidemiology, incidence trends, population, primary cardiac tumors, seer database

## Abstract

Background

Primary malignant cardiac tumors (PMCTs) are considered extremely rare neoplasms, with limited population-level data on incidence patterns and future trends. This study aims to describe the epidemiology of PMCTs in the United States, focusing on three main histologic subtypes, such as sarcomas, lymphomas, and mesotheliomas, and to forecast incidence over the next decade.

Methods

We extracted cases of pathologically confirmed PMCTs diagnosed between 2000 and 2022 from the Surveillance, Epidemiology, and End Results (SEER) 17 Registries database. Incidence rates (IRs) were age-adjusted to the 2000 US Standard Population and stratified by histology, sex, age (<60 vs. ≥60 years), and race. Annual percent change and percentage change were calculated. Time series forecasting was performed using linear regression, ETS, ARIMA, and neural network autoregression models, with model selection based on root mean square error, mean absolute error, mean absolute percentage error, and residual diagnostics. Poisson and quasi-Poisson regression analyses evaluated demographic associations with incidence.

Results

From 2000 to 2022, PMCTs demonstrated distinct subtype-specific trends. Cardiac lymphoma incidence increased steadily, with forecasts predicting a rise from 0.062 per 100,000 in 2023 to 0.080 by 2032. Sarcoma incidence remained stable (~0.008-0.009 per 100,000) but was highly volatile year to year. Mesothelioma incidence was consistently low (<0.001 per 100,000) but projected to increase slightly over time. Age ≥60 years was strongly associated with higher sarcoma and mesothelioma incidence, whereas lymphoma showed no significant age-related difference. Sex and race disparities were evident: males had higher sarcoma and mesothelioma rates, while females had slightly higher lymphoma rates; Asian/Pacific Islander groups exhibited greater volatility in lymphoma incidence, and Black populations had the lowest incidence across subtypes.

Conclusions

PMCTs remain exceptionally rare, but incidence trends vary by histologic subtype. Cardiac lymphoma incidence is rising and expected to continue increasing, whereas sarcoma rates are stable, and mesotheliomas show a modest upward trajectory. These findings highlight the need for improved detection in high-risk groups, the integration of molecular data into registries, and the development of global cardiac tumor collaborations to refine epidemiologic understanding and improve patient care..

## Introduction

Primary malignant cardiac tumors (PMCTs) are exceptionally rare neoplasms. The most comprehensive population-based investigation reported an incidence rate (IR) of ~1.38 per 100,000 persons annually [1], while autopsy studies have estimated frequencies ranging from 0.001% to 0.3% [2,3]. Analyses of large administrative databases also highlight their rarity; for example, only 45,568 benign cardiac neoplasms were identified among >482 million weighted hospital discharges [4]. Malignant tumors constitute roughly one quarter of all primary cardiac neoplasms, with the remainder being benign [2,5].

Recent studies suggest that the incidence of PMCTs is rising. Between 1975 and 2016, the annual percentage change for cardiac sarcomas was 1.7% (p = 0.013) [6], and hospitalizations for PMCTs increased from 1.15 per 100,000 admissions in 2002 to 1.49 per 100,000 in 2017 [7]. Moreover, the median age at diagnosis grew from 57.2 to 62.8 years between 2012 and 2021, despite stable rates of benign cardiac tumors [8].

Among PMCTs, sarcomas are the most frequent and aggressive, representing the majority of cases and often affecting younger adults; five-year survival is as low as 11% [6,9-11]. Primary cardiac lymphomas account for ~1-5% of malignant tumors and usually present in older or immunocompromised patients; prompt chemotherapy can achieve five-year survival up to 34% [6,12-15]. Cardiac mesotheliomas, typically pericardial in origin, constitute ~7-8% of malignant cardiac tumors and are characterized by rapid local invasion and limited therapeutic options [11,16].

Although PMCTs are sporadic, their incidence appears to be rising. Sarcomas predominate, followed by lymphomas and mesotheliomas, with all three subtypes carrying poor prognoses. Despite this data, prior research has largely relied on small series or single-center reports, with limited evaluation of temporal trends across histologic subtypes or of demographic disparities using contemporary population-based data. Forecasts of future incidence are also lacking.

We therefore conducted a Surveillance, Epidemiology, and End Results (SEER)-based analysis to describe incidence trends, histologic distribution, and demographic patterns of PMCTs in the United States from 2000 to 2022 and to forecast incidence through 2032.

## Materials and methods

Data source and extraction

Data for this study were obtained from the National Cancer Institute (NCI)’s SEER database. Specifically, we used the Incidence - SEER Research Data, 17 Registries, Nov 2024 Sub (2000-2022) - Linked to County Attributes - Time Dependent (1990-2023) Income/Rurality, 1969-2023 Counties, released in April 2025 and based on the November 2024 submission. Data extraction was performed using SEER*Stat software (version 9.0).

The SEER database is a comprehensive population-based cancer registry covering approximately 26.5% of the US population, with contributions from multiple registries across diverse geographic areas. It provides detailed information on cancer incidence, patient demographics, tumor characteristics, treatment modalities, and survival outcomes. The database was accessed and processed by AA. It was then interpreted by HK and JA and proofread by SY.

Ethical considerations

AA obtained access to the SEER 17 Registries through the NCI’s SEER*Stat software. As the SEER database is publicly available and does not require a formal accession number, no accession code was issued; all data were accessed in accordance with SEER’s standard data-use agreement.

This study utilized publicly available, de-identified patient data from the SEER database, maintained by the NCI. Because the dataset contains no personally identifiable information, institutional review board approval and informed consent were not required. The analysis adhered to ethical standards for research involving human subjects, maintaining patient confidentiality and data integrity throughout the study. The variables extracted from SEER for this study (sex, age at diagnosis, race, and year of diagnosis) are presented only in aggregated or coded form and cannot, on their own, reveal the identity of any patient.

The SEER database is maintained by the US NCI and contains only de-identified, population-level records. Although the authors are based outside the United States, access to SEER is granted worldwide under its public-use agreement; therefore, no patient records from the authors’ own country were accessed or are publicly available.

Patient and variable selection

All patients included in this analysis were diagnosed between January 1, 2000, and December 31, 2022, with pathologically confirmed PMCTs recorded in the SEER registries. Eligible histologies encompassed soft-tissue sarcomas, lymphomas involving the heart, and mesotheliomas. The primary tumor site was restricted to the heart or pericardium (SEER “Primary Site - Labeled” codes C38.0-C38.3). C38.0 designates the heart, C38.1 the anterior mediastinum, C38.2 the posterior mediastinum, and C38.3 the mediastinum. Cases were excluded if they were classified as benign or borderline behavior, represented secondary or metastatic cardiac tumors, lacked histologic confirmation of malignancy, or had missing demographic variables such as age, sex, or race. Duplicate records and autopsy-only entries without an incidence date were also excluded.

Demographic variables extracted included sex, age at diagnosis, and race (White, Black, American Indian/Alaska Native, or Asian or Pacific Islander). Patients with unknown age or race were excluded from the analysis. Each patient was followed until death or the end of the follow-up period, whichever came first.

In addition to the overall analysis, we conducted subgroup analyses based on the specific histological type, focusing on three primary categories: lymphoma, mesothelioma, and sarcoma. This allowed us to assess incidence trends and demographic patterns for each tumor subtype separately.

Statistical analysis

IRs were calculated per 100,000 population and age-adjusted to the 2000 US Standard Population (age <1 and 90+; Census P25-1130). We examined temporal trends in incidence from 2000 to 2022 overall and within subgroups stratified by sex, race, and age group (<60 years vs. ≥60 years).

To quantify changes over time, we calculated the annual percent change using weighted least squares regression methods. The percentage change was computed as the average yearly change in IRs.

For trend modeling and forecasting, several time series approaches were explored to identify the most accurate method for predicting future IRs. These included linear regression, which models changes over time as a simple trend; the error, trend, seasonal (ETS) framework, which accounts for both systematic and seasonal variations; the autoregressive integrated moving average model, capable of handling complex temporal dependencies; and neural network autoregression (NNAR), which leverages nonlinear patterns in the data. Model performance was compared using the root mean square error (RMSE), mean absolute error (MAE), and mean absolute percentage error (MAPE), and the model demonstrating the best fit was selected for final projections.

Model performance was assessed based on the RMSE, MAE, and MAPE. Residual diagnostics included examination of the autocorrelation function (ACF) and the Ljung-Box test, with nonsignificant p-values indicating adequate model fit. The best-fitting model for each subgroup was used to forecast IRs over the next 10 years.

Additionally, Poisson regression models were applied to assess associations between sex and age ≥60 years with IRs over time. Quasi-Poisson models were employed to evaluate the effect of race over time, accounting for overdispersion. All statistical analyses were performed using SEER*Stat software (version 9.0) and R statistical software for advanced modeling. SEERStat was used because it is the NCI’s standard tool for extracting and analyzing SEER data, ensuring consistent calculation of IRs and trends. R was chosen for advanced time series modeling (ARIMA, NNAR, and ETS) as it offers robust forecasting packages beyond SEERStat’s capabilities.

## Results

IRs (overall, lymphoma, sarcoma, and mesothelioma)

The Three Types of Cancer

**Figure 1:**
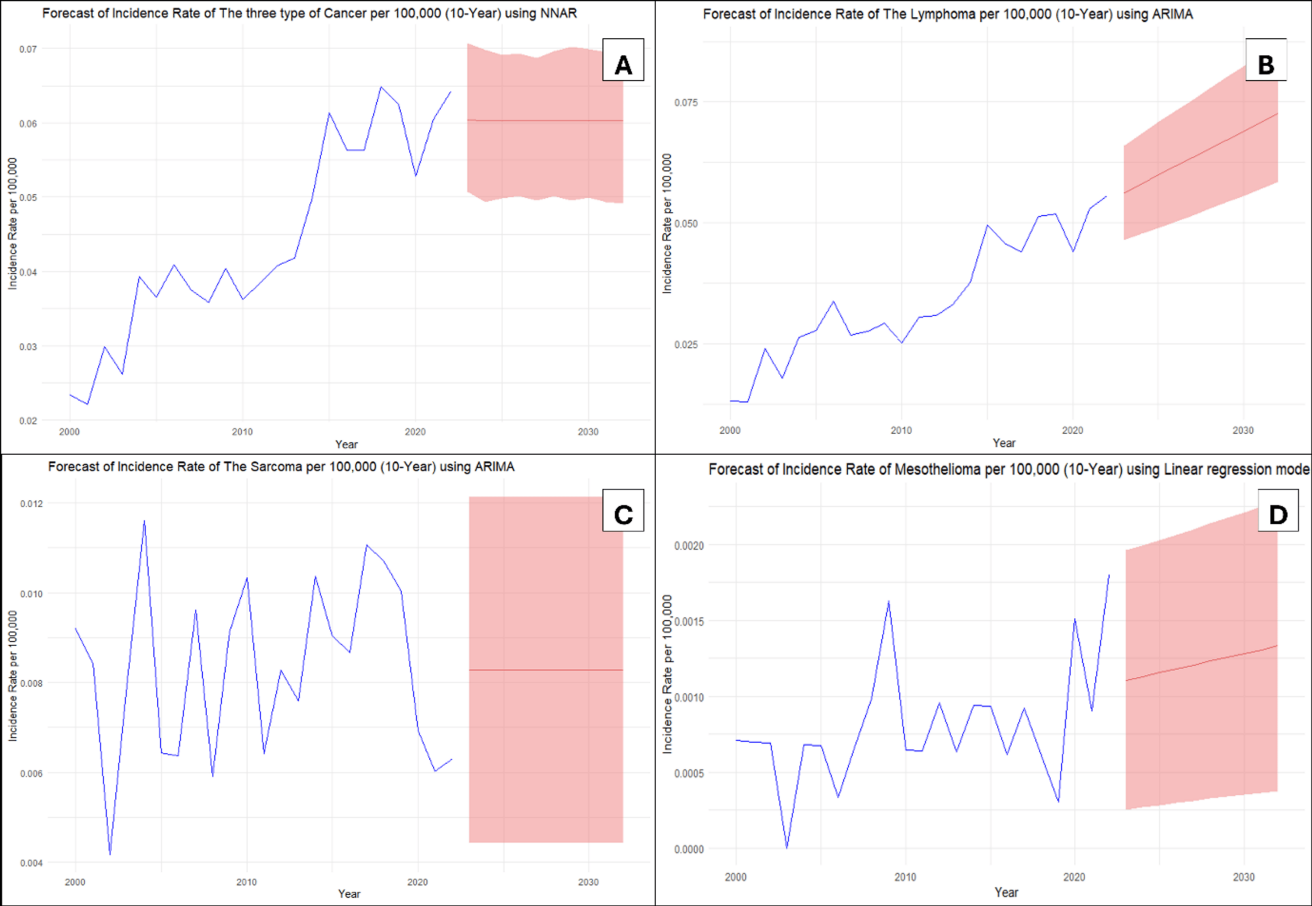
Trends in cancer incidence: (A) Overall. (B) Lymphoma. (C) Sarcoma. (D) Mesothelioma.

The NNAR (1,1) model was selected as the most appropriate model for forecasting because of its good performance in comparison to alternative models like ETS and linear regression. The NNAR (1,1) model showed an acceptable fit within the training dataset, confirmed by reduced residuals measures (RMSE = 0.0047, MAE = 0.0042, and MAPE = 10.4663%). The Ljung-Box test on residuals revealed a nonsignificant p-value of 0.637, confirming the absence of significant autocorrelation and implying that the model accurately captures the underlying structure of the data. The model showed that the IR of the three types of cancer (lymphoma, sarcoma, and mesothelioma) rose over the years from 2000 to 2018, then the trend declined until 2020 and began to increase again in 2021. The predicted values for the next 10 years were stable around a mean of 0.06, ranging between 0.05 and 0.07, as seen in Figure 1. Table 1 provides a summary of the predicted values and the associated prediction intervals.

**Table 1:** Overall IR for each type IR, incidence rate; PI, prediction interval

Year	Predicted IR (95% PI) for the three types	Lymphoma IR (95% PI)	Sarcoma IR (95% PI)	Mesothelioma IR (95% PI)
2023	0.06 (0.05-0.07)	0.06 (0.05-0.07)	0.01 (0.00-0.01)	0.001 (0.000-0.002)
2024	0.06 (0.05-0.07)	0.06 (0.05-0.07)	0.01 (0.00-0.01)	0.001 (0.000-0.002)
2025	0.06 (0.05-0.07)	0.06 (0.05-0.07)	0.01 (0.00-0.01)	0.001 (0.000-0.002)
2026	0.06 (0.05-0.07)	0.06 (0.05-0.07)	0.01 (0.00-0.01)	0.001 (0.000-0.002)
2027	0.06 (0.05-0.07)	0.06 (0.05-0.08)	0.01 (0.00-0.01)	0.001 (0.000-0.002)
2028	0.06 (0.05-0.07)	0.07 (0.05-0.08)	0.01 (0.00-0.01)	0.001 (0.000-0.002)
2029	0.06 (0.05-0.07)	0.07 (0.05-0.08)	0.01 (0.00-0.01)	0.001 (0.000-0.002)
2030	0.06 (0.05-0.07)	0.07 (0.06-0.08)	0.01 (0.00-0.01)	0.001 (0.000-0.002)
2031	0.06 (0.05-0.07)	0.07 (0.06-0.08)	0.01 (0.00-0.01)	0.001 (0.000-0.002)
2032	0.06 (0.05-0.07)	0.07 (0.06-0.09)	0.01 (0.00-0.01)	0.001 (0.000-0.002)

Lymphoma

After closely examining the diagnostic tests and performance measures for each model, the ARIMA (0,1,1) model was chosen for forecasting. The linear regression model had the lowest RMSE (0.00434) of the models evaluated. However, diagnostic testing revealed significant autocorrelation in its residuals (Ljung-Box p = 0.025), which proves that it did not adequately represent the time series structure and goes against one of its fundamental assumptions. With coefficient ma1 = -0.6501, the ARIMA (0,1,1) model had a low RMSE of 0.00466 among all likelihood-based models. Additionally, residual diagnostics demonstrated that the model was adequate. The Ljung-Box test on the residuals gave a nonsignificant p-value of 0.066, indicating the absence of autocorrelation, and the model fit well. This model revealed that lymphoma IR per 100,000 gradually increased over 22 years, and the predicted values for the next 10 years predict an increase in the trend until 2032, as seen in Table 1 and Figure 1.

Sarcoma

After carefully going over each model's performance metrics and diagnostic tests, the ARIMA (0,0,0) model with a nonzero mean was selected for prediction. Out of all the models examined, the linear regression model has the lowest RMSE (0.001917). Its predictor, however, was not statistically significant (p-value = 0.702), making it unreliable for forecasting. Compared to other models, the ARIMA (0,0,0) model had a low RMSE of 0.001924. Furthermore, residual diagnostics showed that the model was sufficient. The residuals' nonsignificant p-value of 0.684 from the Ljung-Box test indicated that there was no autocorrelation. The IR of sarcoma per 100,000 showed high volatility, with fluctuations year-to-year and no clear long-term trend. The predicted values for the next 10 years were stable around a rate of 0.01, as demonstrated in Table 1 and Figure 1.

Mesothelioma

The linear regression model was chosen as the best model for forecasting because it had the least RMSE (0.00036) and the least MAE (0.00027) of all the candidate models, which include ETS and ARIMA. Its primary predictor was statistically significant (p-value = 0.046), confirming a valid, non-random relationship with the forecasted outcome. The Ljung-Box test on the residuals (p-value = 0.838) shows that there is no significant autocorrelation. From 2000 to 2022, the IR of mesothelioma per 100,000 was highly volatile, with sharp and frequent ups and downs. The forecast for the next decade, however, shows a slow but steady increase in the rate around the mean of 0.001, as seen in Table 1 and Figure 1.

IR grouped by sex

Lymphoma

**Figure 2:**
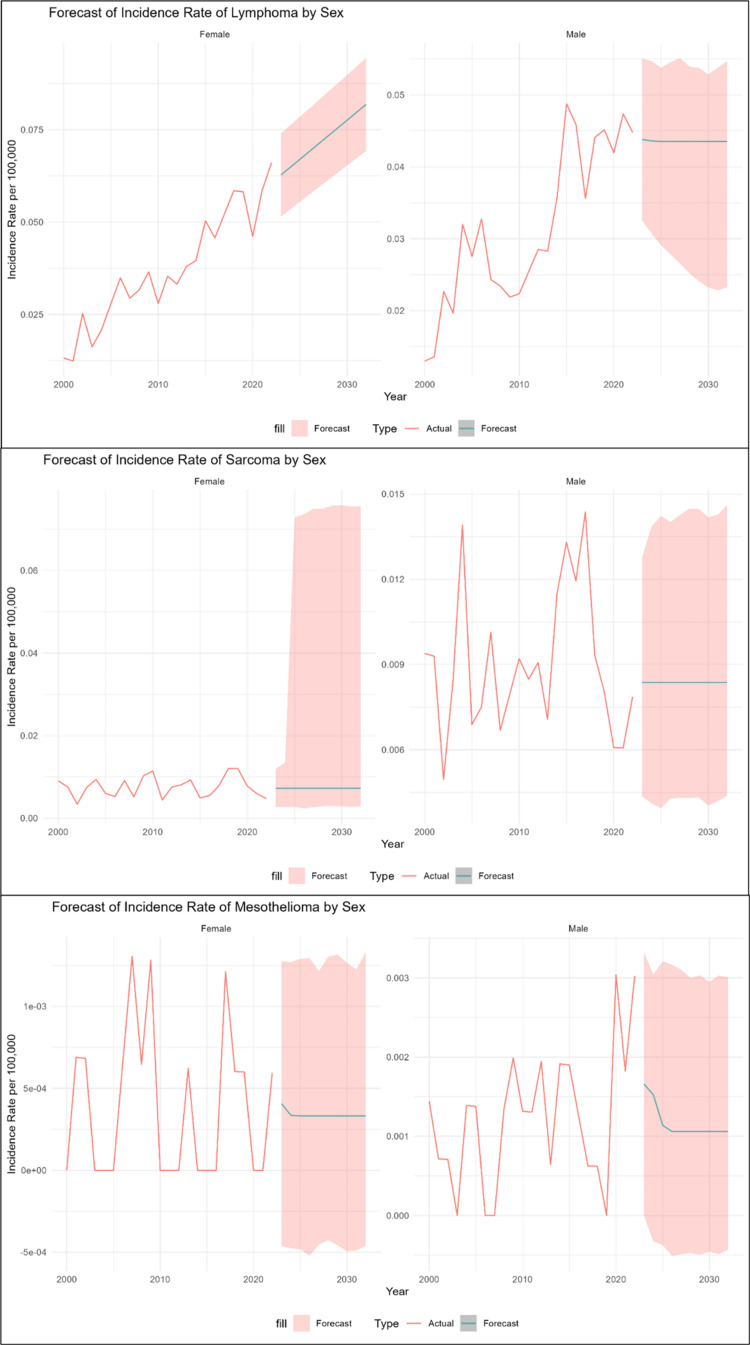
Each type grouped by sex

The chosen model for the male dataset was an NNAR (1,1), with an RMSE of 0.00539, representing an NNAR model that makes use of one lagged input and one hidden node. It averages the outputs of 20 neural networks, each of which has four weights and is set up with a 1-1-1 architecture (1 input, 1 hidden node, 1 output). This framework enables the identification of nonlinear trends while maintaining stability. The model’s sufficiency was confirmed by the nonsignificant result of the Ljung-Box test (p-value = 0.252), which demonstrated that there was no autocorrelation in the residuals. The NNAR model for males revealed that the IR increased gradually between 2000 and 2015, reaching a peak of about 0.049, followed by slight fluctuations and a decrease till 2022. The predicted values from 2023 to 2032 were stable around a mean of 0.0435 with narrow prediction intervals, suggesting minimal uncertainty. For the female dataset, the most effective model was a linear regression model, with an RMSE of 0.0048. The Ljung-Box test p-value of 0.513 indicates that the residuals are uncorrelated, verifying the adequacy of the model. The linear regression model for females showed a stable increase in the IR, with a significant increase noted after 2014, reaching a peak of 0.066 in 2022. The predicted values show a continued upward trend, reaching 0.082 by 2032. The forecasts remain consistent with increasing values (95% PI: 0.069-0.094), showing a steady increase in female IR throughout time, as displayed in Table 2 and Figure 2.

**Table 2:** IRs grouped by sex PIs with negative lower bounds were truncated to 0, as IRs cannot be negative. IR, incidence rate; PI, prediction interval

Year	Male lymphoma	Female lymphoma	Male sarcoma	Female sarcoma	Male mesothelioma	Female mesothelioma
2023	0.044 (0.033-0.055)	0.063 (0.051-0.074)	0.008 (0.004-0.013)	0.007 (0.003-0.012)	0.002 (-0.000-0.003)	0.000 (-0.000-0.001)
2024	0.044 (0.031-0.055)	0.065 (0.053-0.076)	0.008 (0.004-0.014)	0.007 (0.003-0.013)	0.002 (-0.000-0.003)	0.000 (-0.000-0.001)
2025	0.044 (0.029-0.054)	0.067 (0.055-0.078)	0.008 (0.004-0.014)	0.007 (0.003-0.073)	0.001 (-0.000-0.003)	0.000 (-0.000-0.001)
2026	0.044 (0.028-0.055)	0.069 (0.057-0.081)	0.008 (0.004-0.014)	0.007 (0.002-0.074)	0.001 (-0.001-0.003)	0.000 (-0.001-0.001)
2027	0.044 (0.027-0.055)	0.071 (0.059-0.083)	0.008 (0.004-0.014)	0.007 (0.003-0.075)	0.001 (-0.000-0.003)	0.000 (-0.000-0.001)
2028	0.044 (0.025-0.054)	0.073 (0.061-0.085)	0.008 (0.004-0.014)	0.007 (0.003-0.075)	0.001 (-0.000-0.003)	0.000 (-0.000-0.001)
2029	0.044 (0.024-0.054)	0.075 (0.063-0.088)	0.008 (0.004-0.014)	0.007 (0.003-0.076)	0.001 (-0.000-0.003)	0.000 (-0.000-0.001)
2030	0.044 (0.023-0.053)	0.078 (0.065-0.090)	0.008 (0.004-0.014)	0.007 (0.003-0.076)	0.001 (-0.000-0.003)	0.000 (-0.000-0.001)
2031	0.044 (0.023-0.054)	0.080 (0.067-0.092)	0.008 (0.004-0.014)	0.007 (0.003-0.075)	0.001 (-0.000-0.003)	0.000 (-0.000-0.001)
2032	0.044 (0.023-0.055)	0.082 (0.069-0.094)	0.008 (0.004-0.015)	0.007 (0.003-0.076)	0.001 (-0.000-0.003)	0.000 (-0.000-0.001)

Sarcoma

An NNAR model that uses one lagged input and one hidden node was chosen for the male dataset (1,1). Its RMSE is 0.00224. The outputs of 20 neural networks, each with four weights and a 1-1-1 architecture (1 input, 1 hidden node, 1 output), are averaged. This architecture enables the model to detect nonlinear patterns while remaining stable. The Ljung-Box test’s nonsignificant result (p-value = 0.2), which showed that the residuals did not have autocorrelation, validated the model’s sufficiency. The NNAR model for males showed fluctuating IRs from 2000 to 2017, with minor peaks in 2004 and 2017, reaching up to 0.014. After 2017, the IR gradually declined, hitting a low of around 0.006 in 2020 and 2021. Forecasts from 2023 to 2032 remain stable around a mean IR of 0.0084, with relatively narrow prediction intervals, indicating consistent and low variability in future trends. For the female dataset, the chosen model was an NNAR (1,1), with an RMSE of 0.00227, representing an NNAR model that makes use of one lagged input and one hidden node. The outputs of 20 neural networks, each with four weights and a 1-1-1 architecture (1 input, 1 hidden node, 1 output), are averaged. This architecture enables the model to detect nonlinear patterns while remaining stable. The model’s sufficiency was confirmed by the nonsignificant result of the Ljung-Box test (p-value = 0.61), which showed that there was no autocorrelation in the residuals. The model showed variable IRs from 2000 to 2019, with noticeable increases in 2009, 2010, and 2018, peaking at around 0.012. After that, there was a slow decline, and by 2022, the IR had fallen to 0.0048. A stable average IR of 0.0073 is predicted for 2023-2032, while the large and irregular prediction ranges indicate significant uncertainty in future forecasts, as seen in Table 2 and Figure 2.

Mesothelioma

For the male dataset, an NNAR (1,1) with one hidden node and one lagged input was selected. It has a 0.00083 RMSE. Twenty neural networks, each with four weights and a 1-1-1 architecture (one input, one hidden node, and one output), have their outputs averaged. This architecture allows for recognizing nonlinear patterns while maintaining stability. The model’s sufficiency was confirmed by the nonsignificant result of the Ljung-Box test (p-value = 0.986), which indicated that the residuals lacked autocorrelation. The NNAR model for males showed highly variable IRs from 2000 to 2022, with several years dropping to zero and notable peaks around 0.003 in 2020 and 2022. The forecast predicts a slight decrease in the rate until 2026. After this point, the projection flattens out, predicting a stable and constant IR of approximately 0.0011 till 2032, with the prediction intervals suggesting significant uncertainty around this estimate. For the female dataset, the chosen model was an NNAR (1,1), with an RMSE of 0.00043, representing an NNAR model that makes use of one lagged input and one hidden node. The outputs of 20 neural networks, each with four weights and a 1-1-1 architecture (1 input, 1 hidden node, 1 output), are averaged. This architecture enables the model to detect nonlinear patterns while remaining stable. The nonsignificant outcome of the Ljung-Box test (p-value = 0.29), which demonstrated that there was no autocorrelation in the residuals, supported the sufficiency of the model. The NNAR model for females shows a history of very low and sporadic IR from 2000 to 2022, with most years having a rate of zero. This low-level trend will continue, with the IR stabilizing between 2027 and 2032 at consistent, close-to-zero values of about 0.00033. The prediction intervals consistently include zero, indicating that the model expects future rates to remain sporadic and very close to zero, mirroring the trend of rare events throughout history, as seen in Table 2 and Figure 2.

IR grouped by age group

Lymphoma

**Figure 3:**
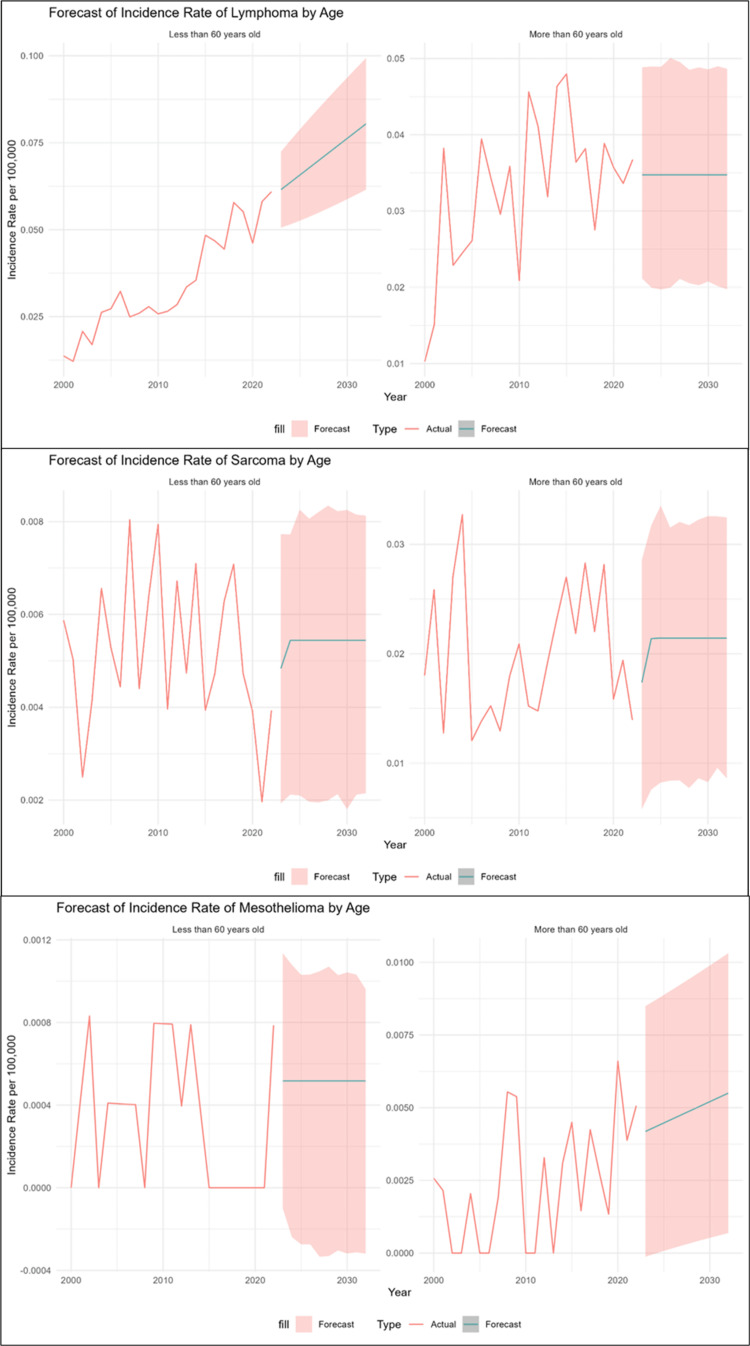
Each type grouped by age group

The best model for the age group <60 years was ARIMA (0,1,1) with drift, with an RMSE of 0.0052. The model’s sufficiency was confirmed by the nonsignificant result of the Ljung-Box test (p-value = 0.176), which demonstrated that there was no autocorrelation in the residuals. For the age group ≥60 years, the most effective model was NNAR (1,1), with an RMSE of 0.0017. The Ljung-Box test p-value of 0.883 indicates that the residuals are uncorrelated, verifying the adequacy of the model. The IR trend in the age group <60 years old was generally lower than in the age group ≥60 years till 2015 but has since exceeded it and is on a clear upward trend. The predicted IR values for the <60 age group are forecasted to be significantly higher than the ≥60 age group, with a projected increase from approximately 0.062 in 2023 to 0.080 in 2032, while the ≥60 group’s forecast remains relatively stable at around 0.035, as displayed in Table 3 and Figure 3.

**Table 3:** IRs grouped by age group PIs with negative lower bounds were truncated to 0, as IRs cannot be negative. IR, incidence rate; PI, prediction interval

Year	Lymphoma <60	Lymphoma ≥60	Sarcoma <60	Sarcoma ≥60	Mesothelioma <60	Mesothelioma ≥60
2023	0.062 (0.051-0.072)	0.035 (0.021-0.049)	0.005 (0.002-0.008)	0.017 (0.006-0.029)	0.001 (-0.000-0.001)	0.004 (-0.000-0.008)
2024	0.064 (0.052-0.076)	0.035 (0.020-0.049)	0.005 (0.002-0.008)	0.021 (0.008-0.032)	0.001 (-0.000-0.001)	0.004 (-0.000-0.009)
2025	0.066 (0.053-0.079)	0.035 (0.020-0.049)	0.005 (0.002-0.008)	0.021 (0.008-0.034)	0.001 (-0.000-0.001)	0.004 (0.000-0.009)
2026	0.068 (0.054-0.082)	0.035 (0.020-0.050)	0.005 (0.002-0.008)	0.021 (0.008-0.032)	0.001 (-0.000-0.001)	0.005 (0.000-0.009)
2027	0.070 (0.055-0.085)	0.035 (0.021-0.050)	0.005 (0.002-0.008)	0.021 (0.008-0.032)	0.001 (-0.000-0.001)	0.005 (0.000-0.009)
2028	0.072 (0.056-0.088)	0.035 (0.021-0.049)	0.005 (0.002-0.008)	0.021 (0.008-0.032)	0.001 (-0.000-0.001)	0.005 (0.000-0.009)
2029	0.074 (0.057-0.091)	0.035 (0.020-0.049)	0.005 (0.002-0.008)	0.021 (0.009-0.032)	0.001 (-0.000-0.001)	0.005 (0.000-0.010)
2030	0.076 (0.059-0.094)	0.035 (0.021-0.049)	0.005 (0.002-0.008)	0.021 (0.008-0.033)	0.001 (-0.000-0.001)	0.005 (0.001-0.010)
2031	0.078 (0.060-0.097)	0.035 (0.020-0.049)	0.005 (0.002-0.008)	0.021 (0.010-0.033)	0.001 (-0.000-0.001)	0.005 (0.001-0.010)
2032	0.080 (0.062-0.099)	0.035 (0.020-0.049)	0.005 (0.002-0.008)	0.021 (0.009-0.032)	0.001 (-0.000-0.001)	0.006 (0.001-0.010)

Sarcoma

We obtained the lowest RMSE values for both age groups using the neural network time series model NNAR (1,1), indicating better prediction accuracy in comparison to other models (ARIMA, ETS, and linear regression). The improved ability of the NNAR (1,1) model to identify data patterns for each age group was demonstrated by the RMSE, which was approximately 0.0014 for the age group under 60 and 0.0057 for the age group over 60. Both groups’ residuals showed no noticeable autocorrelation, according to residual analysis using ACF and the Ljung-Box test (p-values = 1 for age group <60 years and 0.9 for age group ≥60 years). Compared to the younger group, the IR trend was significantly greater and more variable for those 60 and older. The IR for the older group fluctuated significantly, reaching a peak of 0.033 in 2004, and is predicted to stabilize at around 0.021. The group under 60, on the other hand, had a significantly lower IR, peaking at 0.008 in 2007 and expected to stabilize at 0.005. Both groups’ forecasts indicate future stability at these different levels, each with a consistent prediction range, even though their trends were inconsistent, as seen in Table 3 and Figure 3.

Mesothelioma

With an RMSE of 0.0003, NNAR (1,1) was the most effective model for the age group under 60. The nonsignificant Ljung-Box test result (p-value = 0.99), which showed that there was no autocorrelation in the residuals, validated the model’s sufficiency. With an RMSE of 0.0018, the linear regression model proved to be the most successful for the age group of ≥60 years. The model’s sufficiency is confirmed by the Ljung-Box test p-value of 0.26, which shows that the residuals are uncorrelated. There was no apparent trend of one age group continuously outperforming the other because the history IR for both was low and irregular, with rates for both frequently at or near zero. The predicted trends clearly demonstrate a disparity. From roughly 0.004 in 2023 to 0.006 in 2032, the expected IR for the age group ≥60 years old is expected to be much higher and show an upward trend. In contrast, the forecast for the <60 age group stays steady at an insignificant IR of about 0.001, suggesting that the incidence would continuously rise and only increase in the elderly population, as seen in Table 3 and Figure 3.

IR grouped by race

Lymphoma

In forecasting the incidence of lymphoma by racial group, model selection indicated a varied pattern: the linear regression model was the optimal model for the American Indian/Alaska Native and White populations (RMSEs = 0.0145 and 0.0046, respectively), whereas the NNAR excelled for the Black population (RMSE = 0.0058), but ARIMA (2,1,0) with drift was determined to be the most effective model for the Asian or Pacific Islander group (RMSE = 0.0168). The linear regression models effectively represented the level-based structure of American Indian/Alaska Native and White populations, with ACF and residual diagnostics validating adequacy via nonsignificant Ljung-Box test p-values (0.85 and 0.06, respectively), signifying the absence of autocorrelation. The NNAR (2) model fully captured the nonlinear elements for the Black population, with residuals also satisfying the Ljung-Box test (p = 0.45). Also, the ACF and Ljung-Box test for residual analysis validated the statistical adequacy of ARIMA (2,1,0) with drift, with a nonsignificant p-value (0.77). The trend for the Asian or Pacific Islander population was the highest and most unstable, marked by significant peaks in 2016 (IR = 0.120) and 2019 (IR = 0.151), with forecasts suggesting the rate will remain high and irregular. The White population showed a more apparent, more consistent upward trend, starting at a lower IR but steadily increasing over the period and into the forecast.

**Figure 4:**
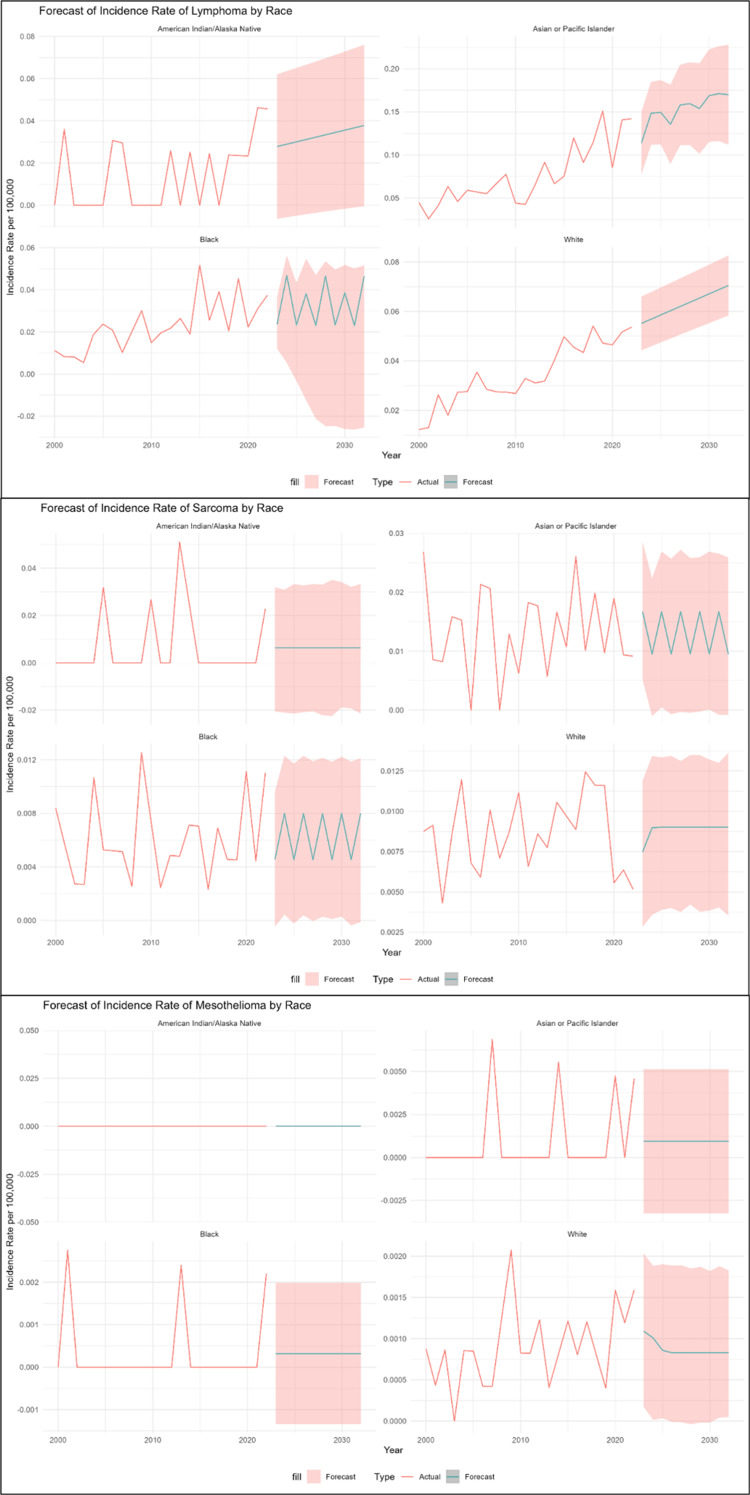
Each type grouped by race

In contrast, the IR for the Black population was highly volatile, with sharp, irregular peaks, such as in 2015 (IR = 0.052), and a forecast that continues this unstable pattern. The American Indian/Alaska Native population displayed the lowest overall trend, which was sporadic, with many years of zero incidence. However, it showed significant peaks in 2021-2022, and it is projected to rise gradually but steadily from its low starting point, as presented in Table 4 and Figure 4.

**Table 4:** Lymphoma grouped by race

Year	Lymphoma White	Lymphoma Black	Lymphoma American Indian/Alaska Native	Lymphoma Asian or Pacific Islander
2023	0.055 (0.044-0.066)	0.024 (0.012-0.037)	0.028 (-0.006-0.062)	0.114 (0.077-0.150)
2024	0.057 (0.046-0.068)	0.047 (0.005-0.056)	0.029 (-0.006-0.064)	0.148 (0.112-0.185)
2025	0.059 (0.047-0.070)	0.023 (-0.003-0.043)	0.030 (-0.005-0.065)	0.149 (0.112-0.187)
2026	0.060 (0.049-0.071)	0.038 (-0.012-0.055)	0.031 (-0.004-0.067)	0.136 (0.089-0.182)
2027	0.062 (0.051-0.073)	0.023 (-0.021-0.047)	0.032 (-0.004-0.068)	0.158 (0.111-0.205)
2028	0.064 (0.052-0.075)	0.047 (-0.025-0.054)	0.033 (-0.003-0.070)	0.160 (0.112-0.207)
2029	0.065 (0.054-0.077)	0.023 (-0.025-0.050)	0.034 (-0.002-0.071)	0.154 (0.101-0.206)
2030	0.067 (0.055-0.079)	0.039 (-0.026-0.052)	0.036 (-0.002-0.073)	0.169 (0.115-0.222)
2031	0.069 (0.057-0.081)	0.023 (-0.026-0.050)	0.037 (-0.001-0.074)	0.171 (0.116-0.226)
2032	0.070 (0.058-0.083)	0.046 (-0.025-0.052)	0.038 (-0.000-0.076)	0.170 (0.112-0.228)

Sarcoma

In predicting sarcoma IR among racial groupings, the NNAR (1,1) model was identified as the best model for all groups. This selection was informed by the minimal RMSE values (0.0022 for the White group, 0.0025 for the Black group, 0.0135 for the American Indian/Alaska Native group, and 0.0057 for the Asian or Pacific Islander group). Residual analysis validated the sufficiency of all models, with ACF and Ljung-Box test p-values demonstrating the absence of substantial autocorrelation: 0.73 (American Indian/Alaska Native), 0.6 (Asian or Pacific Islander), 0.3 (Black), and 0.85 (White). The American Indian/Alaska Native population had the most irregular pattern, with many years with zero incidence and the highest peak of any group (IR = 0.051 in 2013). Yet, its prediction shows a substantial shift to a low, steady rate. With notable peaks in 2000 (IR = 0.027) and 2016 (IR = 0.026), the Asian or Pacific Islander population also showed an irregular trend and is expected to continue to fluctuate in the future. The Black population’s IR was also volatile, peaking at about 0.013 in 2009, and is expected to continue this unstable, fluctuating trend at a generally lower level. The White population, on the other hand, exhibited the most consistent trend, continuously varying within a relatively small range and expected to maintain a constant IR of roughly 0.009.

Mesothelioma

Model selection revealed a varied pattern in predicting the incidence of mesothelioma by racial group: NNAR performed well for the White population (RMSE = 0.0005). In contrast, the ARIMA (0,0,0) with a nonzero mean model was the best model for the Asian or Pacific Islander and Black populations (RMSEs = 0.0021 and 0.0008, respectively). However, analysis of the American Indian/Alaska Native subgroup showed no reported cases of mesothelioma during the entire study period from 2000 to 2022; as a result, the annual IR for this population was consistently 0 per 100,000. Because of the complete lack of variance in this time series, the proposed forecasting models (ARIMA, ETS, and NNAR) could not be statistically fitted, resulting in a sustained, observed IR of zero for this cohort. The ARIMA models effectively expressed the level-based structure of Asian or Pacific Islander and Black populations, with ACF and residual diagnostics validating sufficiency via nonsignificant Ljung-Box test p-values (0.72 and 0.97, respectively), indicating that the absence of autocorrelation in the population’s nonlinear elements was successfully captured by NNAR (1,1). The residuals also passed the Ljung-Box test (p = 0.64). Although consistently low, the White population’s IR was the most observed, exhibiting a persistent but irregular trend that is expected to stabilize at a very low rate of about 0.001. Black and Asian or Pacific Islander groups, on the other hand, showed very irregular trends, with IRs of zero in the majority of years. Whereas the Black population’s peaks were lower, the Asian or Pacific Islander group had the most significant single peak in the data (IR = 0.007 in 2007); both groups are expected to stabilize at a very low rate.

Poisson regression

Lymphoma

The Poisson regression analysis for lymphoma indicated that an age of 60 years or older was not associated with a statistically significant difference in incidence compared to the younger group (IRR = 0.913, 95% CI: 0.825-1.009, p = 0.076). However, this model revealed a significant increasing trend in incidence over time (IRR = 1.054, 95% CI: 1.048-1.061, p < 0.001). In a second Poisson model assessing sex, males exhibited a statistically significant lower IR relative to females (IRR = 0.841, 95% CI: 0.777-0.910, p < 0.001), while the substantial increase in incidence over time was again observed (IRR = 1.054, 95% CI: 1.047-1.060, p < 0.001). A third analysis, using a quasi-Poisson model, evaluated the impact of race with Asians as the reference category. The results indicated significantly lower IRs for all other groups compared to the reference group: American Indian/Alaska Native individuals (IRR = 0.191, 95% CI: 0.099-0.329, p < 0.001), Black individuals (IRR = 0.299, 95% CI: 0.249-0.359, p < 0.001), and White individuals (IRR = 0.449, 95% CI: 0.396-0.511, p < 0.001). The temporal variable in this model also confirmed a significant rise in incidence over the study period (IRR = 1.053, 95% CI: 1.046-1.060, p < 0.001).

Sarcoma

The Poisson regression analysis for sarcoma by age group indicated that persons in the older age group (>60) had a significantly increased incidence, reflected by an estimated IR ratio (IRR) of 3.848 (95% CI: 3.269-4.528, p < 0.001). Time was not a significant variable in this model (IRR = 0.998, 95% CI: 0.986-1.011, p = 0.785). In an independent model accounting for sex and time, the IRR for males was 1.189 (95% CI: 1.011-1.397, p = 0.036), representing a statistically significant increase in incidence compared to females. Likewise, time was not a substantial factor in this second model (IRR = 1.003, 95% CI: 0.990-1.015, p = 0.675). In a third model assessing the correlation between race and sarcoma incidence, quasi-Poisson regression indicated that, relative to Asians (reference group), Black individuals exhibited a significantly lower IRR (IRR = 0.455, 95% CI: 0.314-0.662, p < 0.001), as did White individuals (IRR = 0.643, 95% CI: 0.489-0.864, p = 0.003). The American Indian/Alaska Native group did not demonstrate a statistically significant association (IRR = 0.533, 95% CI: 0.208-1.131, p = 0.141). The year variable was nonsignificant in the race model (IRR = 1.003, 95% CI: 0.991-1.015, p = 0.639).

Mesothelioma

The Poisson regression model for mesothelioma by age group revealed a statistically significant correlation, with persons aged ≥60 years exhibiting an IRR of 7.683 (95% CI: 4.472-13.642, p < 0.001) in comparison to the younger reference group. This signifies that the IR among older adults is more than 7.5 times higher, highlighting age as a significant risk factor. In this model, time was not a significant predictor (IRR = 1.026, 95% CI: 0.986-1.069, p = 0.210). In a distinct model evaluating sex and time, male sex was significantly correlated with mesothelioma incidence, with an IRR of 3.237 (95% CI: 1.822-6.126, p < 0.001). Time was also nonsignificant in the second model (IRR = 1.032, 95% CI: 0.992-1.075, p = 0.119). No cases of mesothelioma were reported among the American Indian/Alaska Native population. This group was therefore excluded from the regression analysis. A third model, which included the remaining racial groups and year, employed a quasi-Poisson regression. In comparison to Asians (reference group), the IRR for Black persons was 0.313 (95% CI: 0.078-1.155, p = 0.085) and for White persons was 0.904 (95% CI: 0.410-2.496, p = 0.823), with neither result indicating a lack of statistical significance. The year variable was also not significant in this model (IRR = 1.033, 95% CI: 0.999-1.070, p = 0.066).

## Discussion

Incidence trends in cardiac tumors

Our analysis of SEER data for malignant cardiac tumors revealed varying incidence trends among the three histological types: primary cardiac sarcomas, mesotheliomas, and lymphomas. Overall, the incidence of these uncommon tumors rose steadily between 2000 and the late 2010s, followed by a slight decline between 2019 and 2020 and a subsequent rise in 2021. Primary cardiac lymphomas showed the most pronounced long-term increase among the subtypes, with the incidence gradually increasing over the course of the research. This increasing trend is projected to continue over the next decade, with estimates rising from roughly 0.06 per 100,000 in 2023 to 0.07 per 100,000 in 2032.

While there was no discernible rising or decreasing trend, cardiac sarcomas demonstrated greater year-to-year fluctuation compared to other subtypes. Over the next ten years, the forecast shows a consistent rate of 0.01 per 100,000, although their incidence is higher than that of mesotheliomas. During the monitoring period, cardiac mesotheliomas, which are mainly pericardial in origin, were extremely uncommon. Projections indicate the rate will remain the same, 0.001 per 100,000 from 2023 to 0.001 per 100,000 by 2032, although historical incidence exhibited noticeable swings. Our analyses indicate that cardiac mesothelioma remains exceedingly uncommon in the overall population, with forecasts stabilizing around 0.001 per 100,000 through 2032. However, age-stratified projections show a clear increase among individuals ≥60 years, with incidence expected to rise from 0.004 in 2023 to ~0.006 by 2032 (Table 3). This divergence is consistent with our Poisson regression, which identified age ≥60 years as a strong risk factor (IRR = 7.68; 95% CI: 4.47-13.64). The excess in older adults likely reflects the long latency of asbestos-related disease and cumulative somatic mutations, whereas the persistently low rates in younger cohorts suggest minimal ongoing exposures.

These trends are generally in line with earlier studies that described primary cardiac tumors as sporadic cancers exhibiting diverse etiologies and clinical courses [17]. An aging population with weaker immune systems, enhanced diagnostic capabilities, and increased use of advanced cardiac imaging could all contribute to the ongoing increase in the incidence of cardiac lymphoma [18]. On the other hand, sarcoma and mesothelioma rates are pretty stable, which is consistent with their aggressive biology, brief clinical course, and stable environmental risk factors over time [19,20].

Globally, the IRs of cardiac mesothelioma in the United States are relatively stable when compared with regions with less comprehensive asbestos regulation. For instance, mesothelioma incidence, especially pleural, is still on the rise in areas of Asia where asbestos usage is still prevalent, such as China, India, and some countries in Southeast Asia. This trend is expected to continue in the upcoming decades as occupational exposures become apparent after extended latency periods [21]. Despite being extremely uncommon globally [22], pericardial mesothelioma trends probably resemble these pleural patterns, indicating that future global incidence may differ significantly from that in nations like the United States that were among the first to outlaw asbestos.

Demographic disparities and risk factors

Age and Sex

The incidence of mesotheliomas and cardiac sarcomas was significantly influenced by age. The incidence of these malignancies was much higher in people over 60 than in people under 60. Because asbestos-related cancers have a lengthy latency, older adults had an IRR for mesotheliomas that was more than seven times higher than that of younger people [23,24]. In line with the cumulative effects of environmental exposures and somatic mutations over time, the incidence of sarcoma was likewise almost four times greater in older persons [20]. However, there was no statistically significant difference in the incidence of cardiac lymphomas between age groups, which probably reflects a subset of younger patients who had undergone post-transplant therapy or HIV/AIDS-related immunosuppression [25].

Differences based on sex were also noted. In line with established occupational exposure patterns for asbestos and other carcinogens, males were more likely to develop cardiac sarcomas and mesotheliomas [24]. Similar to trends in pleural mesothelioma, the incidence of mesothelioma in men was more than three times higher than in women. In contrast, compared to systemic non-Hodgkin lymphoma trends, females demonstrated moderately higher IRs of cardiac lymphoma in recent years. These findings may indicate sex-specific differences in immune surveillance or diagnostic detection patterns.

Race and Ethnicity

Although there were noticeable racial and ethnic differences, these findings require cautious interpretation due to the modest/limited number of cases. For some subtypes, particularly lymphoma, Asian or Pacific Islander (API) populations had the highest and most variable IRs, with sporadic increases above 0.12 per 100,000. While white populations demonstrated the highest baseline IRs overall, their growth patterns showed more gradual, linear increases over time compared to other racial groups. American Indian/Alaska Native populations had limited data, with particular subtypes, like mesothelioma, showing no documented instances during the study period, while Black populations consistently had the lowest IRs for all three tumor types.

Although the exact cause of these discrepancies is unknown, possible causes include genetic predisposition, variations in environmental exposure, access to healthcare, and biases in diagnostic ascertainment [17]. For instance, White and Asian or Pacific Islander males have historically shown higher occupational asbestos exposure levels compared to other racial groups [24], potentially explaining the differences in mesothelioma IRs.

Immunosuppression and Other Risk Factors

Immunosuppression represents a well-established risk factor for primary cardiac lymphoma development, particularly heart-related Epstein-Barr virus (EBV)-driven lymphoproliferative diseases. These occur most frequently in immunocompromised populations, including patients with HIV/AIDS, post-transplant immunosuppression regimens, or long-term immunosuppressive therapies [25]. Radiation-related angiosarcoma of the heart has been connected with previous chest irradiation in sarcomas [20]. Additionally, the risk of cardiac sarcomas, especially angiosarcoma, is increased in rare genetic cancer syndromes such as Li-Fraumeni syndrome [26].

Their diverse biology probably causes the incidence of sarcomas (0.009/100,000): radiation-associated subtypes rely on fixed prior exposures, whereas TP53-mutant angiosarcomas might develop de novo through random somatic mutations [27]. In contrast, the extended latency after asbestos exposure and the gradual introduction of occupational restrictions in the United States in the late 20th century may be contributing factors to the little increase in the prevalence of cardiac mesothelioma [28]. The recent increase may be the tail end of the exposure cohort from the 1970s and 1980s, as mesothelioma cases diagnosed today may reflect exposures from those years due to a latency period that frequently exceeds 30 years.

Methodology and forecasting strengths

This study’s use of both ARIMA and NNAR models to evaluate temporal patterns and provide forecasts was one of its strongest points. In epidemiological research, ARIMA models are frequently used to identify and forecast linear patterns in time series data [29]. On the other hand, nonlinear patterns and irregular fluctuations can be captured by NNAR models, which is especially important for uncommon malignancies with irregular incidence patterns [30]. Fit statistics (RMSE, MAE, and MAPE) and residual diagnostics were used to guide the model selection process, guaranteeing that the models selected offered both biological plausibility and statistical robustness.

The dual-model approach allowed a nuanced interpretation of trends across tumor types and subgroups. For example, NNAR effectively modeled the irregular incidence of mesothelioma, while ARIMA captured the steady upward trajectory of lymphoma. This methodology strengthens the validity of our forecasts, which suggest continued modest increases in lymphoma, stable sarcoma rates, and a slow rise in mesothelioma over the next decade.

Strengths and limitations

The principal strengths of this study include the use of an extensive, population-based cancer registry (SEER), long-term follow-up, and advanced statistical modeling. This approach allowed for robust estimates of incidence trends and subgroup analyses despite the rarity of these tumors. The combination of ARIMA and NNAR modeling provided complementary perspectives, enhancing forecast reliability.

For volatile populations, like the Asian or Pacific Islander population with cardiac lymphoma, where IRs varied significantly from year to year, the value of NNAR modeling was obvious. By utilizing nonlinear correlations in the data, NNAR produced stable projections, while traditional linear models ran the danger of overfitting or misdescribing such irregular patterns. However, because these tumors are so uncommon, small sample sizes continue to limit analyses that are unique to race and ethnicity. In practice, this means that a single extra case can disproportionately impact anticipated trajectories and computed IRs in a small cohort. This statistical fragility highlights the necessity for bigger, pooled datasets and should be specifically noted when interpreting findings of racial difference.

Several limitations must be acknowledged. The rarity of primary cardiac tumors results in small case numbers, limiting statistical power and the precision of subgroup estimates. SEER lacks detailed exposure histories, immunosuppression status, and molecular data, which limits the ability to assess causation. Diagnostic misclassification is possible, particularly for distinguishing primary from metastatic cardiac tumors. Finally, forecasts assume the continuation of historical trends and may not account for sudden changes in exposure patterns, diagnostic practices, or therapeutic advances.

Future directions

In the future, including genetic and molecular profiling in population-based registries may significantly advance our understanding of etiology. Systematic screening for TP53 mutations may help identify the role of Li-Fraumeni syndrome and other genetic predispositions in cardiac sarcomas. EBV status may help distinguish between spontaneous and immunodeficiency-associated cardiac lymphomas. Additionally, such data would allow trend analyses to be more finely stratified. On a larger scale, the creation of a global registry for cardiac tumors that would combine cases from various socioeconomic and geographic backgrounds would enable more accurate incidence estimation, support international cooperation on prevention and early detection tactics, and validate or disprove apparent racial and regional disparities.

## Conclusions

This SEER-based study demonstrates distinct incidence patterns among PMCTs in the United States: sarcomas remain stable but fluctuate year to year, lymphomas are steadily rising, and mesotheliomas, while rare overall, are increasing among older adults. These findings underscore the need for heightened awareness and early recognition in high-risk groups, systematic integration of histologic and molecular data into population registries, and collaborative research to refine surveillance and management strategies for these uncommon tumors.
